# Reply to “Enhancing PCI strategies for severely calcified coronary lesions: gaps and new directions”

**DOI:** 10.1007/s00380-024-02495-2

**Published:** 2024-11-29

**Authors:** Yoriyasu Suzuki

**Affiliations:** 1 Department of Cardiology, Aichi Medical University, 1-1 Yazakorimata, Nagakute, Aichi 480-1195 Japan; 2 Department of Cardiology, Nagoya Heart Center, 1-1-14 Sunadabashi Chikusa-ku, Nagoya, Aichi 461-0045 Japan

Dear Editor,

We appreciate the thoughtful and constructive comments provided by Rajakumar et al. regarding our recent article, *"Clinical Outcomes of Percutaneous Coronary Intervention for Severely Calcified Coronary Lesions: Comparison Between the Morphologies of Severely Calcified Coronary Lesions."* We are grateful for the opportunity to address their feedback and provide clarification on the points raised.

## Imaging modalities

We acknowledge the limitations of intravascular ultrasound (IVUS) in distinguishing different forms of calcification, such as calcified nodules and eccentric calcifications. Optical coherence tomography (OCT) indeed offers a higher resolution that could enhance visualization of calcification characteristics, as suggested by Rajakumar et al. However, the use of OCT was restricted due to patient selection criteria and the practical challenges of using OCT to image complex calcified lesions in a real-world clinical setting. We agree that further studies incorporating OCT alongside IVUS may provide a more comprehensive understanding of calcification and improve treatment strategies.

## Alternative strategies for IVUS failure

Rajakumar et al. emphasize that approximately 60% of lesions failed to pass the IVUS catheter. We would like to clarify that the IVUS catheter did not initially pass the target lesion, requiring ablation with an atherectomy device before IVUS evaluation. In routine clinical practice, lesions that do not allow IVUS passage are likely to pose similar challenges for OCT. At our institution, we aim to evaluate calcified lesions with intravascular imaging whenever possible before formulating the PCI strategy. If IVUS passage is unsuccessful, we attempt balloon dilation. When balloon passage proves difficult, we use a Rotablator. Approximately 60% of the patients in this study had such complex lesions.

## Device selection and efficacy

We acknowledge the absence of a detailed subgroup analysis comparing the efficacy of rotational versus orbital atherectomy as a limitation of our study. Our research aimed to provide a broad overview of PCI outcomes across various calcification morphologies, rather than focusing on device-specific outcomes. We recognize the importance of subgroup analysis and plan to include this in future research to better tailor device selection based on specific lesion characteristics.

## Stent symmetry index (SSI)

We agree with Rajakumar et al. that achieving optimal stent symmetry is crucial for reducing restenosis and stent thrombosis. While our study highlighted the association between lower SSI and higher rates of device-oriented composite endpoints (DoCE), it did not focus on specific techniques to improve SSI. In eccentric lesions with a calcium arc of less than 270°, lumen enlargement can be achieved by stretching the non-calcified quadrant. However, this does not result in significant calcium modification, including calcium fractures, as it primarily involves stretching the non-calcified vessel wall. The use of atherectomy devices, particularly orbital atherectomy (OA), is expected to achieve significant calcium modification in eccentric lesions. Nevertheless, its effectiveness depends on factors, such as wire bias, calcium distribution, and lumen dimensions [1–3]. Thus, OA may not be universally effective for all lesion types. We appreciate the authors' suggestion to consider advanced lesion preparation and post-dilation strategies, and we believe that these points merit further investigation in future studies.

## Calcium fracture techniques

We recognize the importance of calcium fractures in improving vessel compliance and stent expansion. Our study identified calcium fractures as predictors of successful PCI outcomes, but did not provide detailed guidance on achieving this. At the time of our study, intravascular lithotripsy (IVL) was not yet available in Japan, which limited our ability to explore this technique. Instead, we frequently used scoring balloons, particularly in concentric calcified lesions, with calcium fractures observed in nearly 80% of cases. Eccentric and calcified nodule lesions were treated less frequently, resulting in fewer observed calcium fractures. We agree with Rajakumar et al. that methods such as IVL and advanced atherectomy techniques are valuable in promoting calcium modification. Ziad et al. reported no significant difference in residual area stenosis, stent area, stent expansion, or acute gain between eccentric and concentric lesions treated with IVL [4]. We intend to explore these techniques in future studies to better understand their role in optimizing PCI outcomes for calcified lesions.

## Conclusion

We are grateful to Rajakumar et al. for their insightful feedback, which has not only validated many of our findings but also highlighted areas for future improvement. Incorporating the constructive feedback provided, we aim to expand our use of imaging techniques and advanced lesion preparation strategies in our future studies. We look forward to further collaborative efforts in refining PCI strategies for severely calcified lesions, particularly through the integration of alternative imaging modalities, enhanced lesion preparation techniques, and improved safety measures.
